# ERCC1 expression as a predictive marker of squamous cell carcinoma of the head and neck treated with cisplatin-based concurrent chemoradiation

**DOI:** 10.1038/sj.bjc.6604464

**Published:** 2008-07-01

**Authors:** H J Jun, M J Ahn, H S Kim, S Y Yi, J Han, S K Lee, Y C Ahn, H-S Jeong, Y-I Son, J-H Baek, K Park

**Affiliations:** 1 Division of Hematology-Oncology, Department of Medicine, Samsung Medical Center, Sungkyunkwan University School of Medicine, Seoul 135-710, Korea; 2 Department of Pathology, Samsung Medical Center, Sungkyunkwan University School of Medicine, Seoul 135-710, Korea; 3 Department of Pathology, Kangwon National University Hospital, Kangwon National University School of Medicine, Kangwon-do 200-947, Korea; 4 Department of Radiation Oncology, Samsung Medical Center, Sungkyunkwan University School of Medicine, Seoul 135-710, Korea; 5 Department of Head and Neck Surgery, Samsung Medical Center, Sungkyunkwan University School of Medicine, Seoul 135-710, Korea

**Keywords:** ERCC1, squamous cell carcinoma, head and neck cancer, cisplatin, concurrent chemoradiation

## Abstract

The excision repair cross-complementation group 1 (ERCC1) enzyme plays a rate-limiting role in the nucleotide excision repair pathway and is associated with resistance to platinum-based chemotherapy. The purpose of this study was to evaluate the role of ERCC1 expression as a predictive marker of survival in patients with locally advanced squamous cell carcinoma of the head and neck (SCCHN) treated with cisplatin-based concurrent chemoradiotherapy (CCRT). ERCC1 expression was assessed by immunohistochemical staining. The median age of the 45 patients analysed was 56 years (range 27–75 years), and 82% were men; 73% of all specimens showed high expression of ERCC1. The overall tumour response rate after CCRT was 89%. The median follow-up was 53.6 months (95% CI, 34.5–72.7 months). The 3-year progression-free survival (PFS) and overall survival (OS) rates were 58.7 and 61.3%, respectively. Univariate analyses showed that patients with low expression of ERCC1 had a significantly higher 3-year PFS (83.3 *vs* 49.4%, *P*=0.036) and OS (91.7 *vs* 45.5%, *P*=0.013) rates. Multivariate analysis showed that low expression of ERCC1 was an independent predictor for prolonged survival (HR, 0.120; 95% CI, 0.016–0.934, *P*=0.043). These results suggest that ERCC1 expression might be a useful predictive marker of locally advanced SCCHN in patients treated with cisplatin-based CCRT.

Squamous cell carcinoma of the head and neck (SCCHN) accounts for over 6% of all malignancies (Globocan, 2000) and most patients present with locally advanced disease. Recent trials reported improved locoregional control and overall survival (OS) by adding chemotherapy to radiotherapy concurrently (Brizel *et al*, 1998; Forastiere *et al*, 2003). The value of concurrent chemoradiotherapy (CCRT) is counterbalanced by increased therapy-associated complication rates. Therefore, identifying molecular markers that can predict which patients benefit from CCRT is crucial in the management of patients with locally advanced SCCHN.

Cisplatin is the backbone of the chemotherapy regimen as a component of CCRT in the treatment of locally advanced SCCHN. Its main cytotoxic activity is based on the formation of DNA adducts, which cause inter- and intrastrand cross-linking. The nucleotide excision repair pathway is considered as one of the most important pathways that guard the integrity of the genome by recognising and removing a variety of DNA cross-links caused by cisplatin or radiation (Dabholkar *et al*, 1994; Murray and Rosenberg, 1996). Excision repair cross-complementation group 1 (ERCC1) plays a key role in nucleotide excision repair and in removing platinum-induced DNA adducts (Johnson *et al*, 2001). A correlation between increased ERCC1 expression with resistance to cisplatin or with poor survival has been reported for several tumours including SCCHN (Metzger *et al*, 1998; Shirota *et al*, 2001; Joshi *et al*, 2005; Handra-Luca *et al*, 2007). Polymorphisms in metabolic enzymes and in DNA repair genes are related to the treatment response in lung (Rosell *et al*, 2002), cervical (Britten *et al*, 2000), colon (Viguier *et al*, 2005), and other (Yu *et al*, 1997; Zhou *et al*, 2004) cancers.

Radiotherapy also injures genetic material and increases apoptosis in tumour cells. Genetic polymorphisms in DNA repair genes may significantly influence the response to radiotherapy in stage I–II head and neck cancer (Carles *et al*, 2006).

The purpose of this study was to evaluate whether the immunohistochemical expression status of ERCC1 can predict the tumour response and cancer-specific survival in patients with locally advanced SCCHN being treated with cisplatin-based CCRT.

## Materials and methods

### Patients and treatment

A total of 60 patients with histologically or cytologically proven locally advanced SCCHN were treated with CCRT between 1995 and 2005 at Samsung Medical Center (Seoul, Korea). Forty-five samples adequate for analysis of ERCC1 expression were enrolled in this study. The performance status was ECOG 0–1. Inclusion criteria included patients with adequate bone marrow, liver, and renal function. None of the patients had prior radiotherapy or chemotherapy.

The chemotherapy regimens comprised cisplatin with or without 5-fluorouracil or taxane. The radiation dose was 72 Gy over 7 weeks (2 Gy day^−1^, 5 fractions per week). Pretreatment evaluation included the patient's history, physical examination, performance status, chest X-ray, complete blood count, blood chemistry, and computed tomography (CT) scan or magnetic resonance imaging (MRI) of the head and neck. The response to CCRT was assessed according to the World Health Organization (WHO) criteria. Patients were evaluated by CT scan or MRI of the head and neck every 3 months for 2 years, and then every 6 months thereafter. Approval was obtained from the Institutional Review Boards, according to legal regulations.

### Immunohistochemical staining for ERCC1

Formalin-fixed paraffin-embedded tissue blocks were sectioned at 4 *μ*m thickness. The tissue sections were deparaffinised in xylene and then rehydrated in serial-graded alcohol. Excision repair cross-complementation group 1 antigen retrieval comprised heating in 10 mM citrate buffer at pH 6.0 in a microwave (15 min, 700 W) and cooling at room temperature for 20 min. The sections were washed in Tris-buffered saline (TBS), and the slides were pre-incubated with 5% normal blocking solution (goat serum) for 10 min to reduce nonspecific binding. The slides were incubated at room temperature with mouse monoclonal anti-ERCC1 (8F1; Neomarkers, Fremont, CA, USA) (Wachters *et al*, 2005; Olaussen *et al*, 2006; Handra-Luca *et al*, 2007) at a dilution of 1 : 200 overnight in a humidified chamber. The primary antibody was visualised with an avidin–biotin complex (ABC) system (Dako, Carpinteria, CA, USA). The slides were washed in TBS, the relevant biotinylated goat anti-mouse IgG diluted at 1 : 100 was added, and the slides were incubated for 20 min at room temperature. The sections were washed again in TBS and incubated for 10 min in a solution of streptavidin–ABC–horseradish peroxidase diluted at 1 : 100. Colour was developed by adding 3,3′-diaminobenzidine tetrahydrochloride (Immunotech, Cedex, France). Finally, the sections were counterstained with Mayer's haematoxylin.

### Evaluation of ERCC1 expression

Two pathologists (JH and SKL), who were unaware of the clinical data, evaluated the ERCC1 staining independently under a light microscope at a magnification of × 400. The pathologists recorded whether tumour or stromal cells expressed ERCC1. The staining intensity was graded on a scale of 0–3; endothelial cells were used as the internal reference and assigned an intensity of 2. Five images of representative areas were acquired for each specimen. A total of 500–1500 positive or negative tumour nuclei per specimen were counted manually on a computer screen. The percentage of positive nuclei was calculated for each specimen, and a proportion score was assigned (0 if 0%, 0.1 if 1–9%, 0.5 if 10–49%, and 1.0 if ⩾50%). The proportion score was multiplied by the staining intensity to obtain a final semi-quantitative *H* score. The median value of the *H* score was chosen as the cutoff point for separating low and high levels of ERCC1 expression, as described previously (Olaussen *et al*, 2006).

### Statistical analysis

The baseline characteristics of the low and high levels of ERCC1 expression groups of patients were compared using the Fisher's exact test for discrete variables and the Mann–Whitney *U*-test for continuous variables. The OS duration was calculated from the first day of the CCRT until the date of death or the latest documented follow-up. Progression-free survival (PFS) was calculated from the first day of the CCRT to the day when the disease progression was recognised or the day of the last follow-up visit. Survival rates were estimated using the Kaplan–Meier method. The prognostic value of ERCC1 status was studied using Cox models adjusted for known prognostic factors, such as age, tumour location, and TNM stage. All reported *P*-values are two-sided and *P*<0.05 was considered significant.

## Results

### Patient characteristics

The median age of the patients was 56 years (range 27–75 years), and 82% were men. Eight patients had stage III, 30 had stage IVA, and seven had stage IVB disease. The most common sites were the oropharynx (15 out of 45, 33%), followed by the hypopharynx (12 out of 45, 27%) (Table 1). Of the 45 patients who initially entered into the study, 17 patients were treated with cisplatin only (38%); 6 with 5-fluorouracil plus cisplatin (13%); and 22 with taxane plus cisplatin (49%). The median dose of administered cisplatin was 225 mg m^−2^ (range 60–300 mg m^−2^) and the median radiation was 6660 cGy (range 3960–7200 cGy). Twenty patients received more than 70 Gy of radiation dose and 38 patients completed the planned chemotherapy.

### Clinical–pathological data and ERCC1 expression

**Figure 1:**
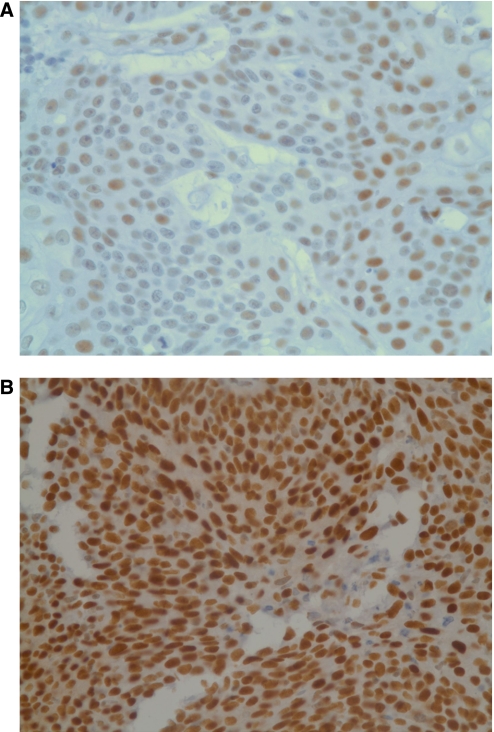
Representative examples of ERCC1 immunostains. (**A**) *H* score <2. (**B**) *H* score ⩾2. Original magnification, × 400.

Excision repair cross-complementation group 1 expression was localised to the nucleus, and the median *H* score for SCCHN tumours was 2.0 (Figure 1). Thirty-three (73%) tumours with *H* score ⩾2.0 were defined as having high expression of ERCC1.

The clinical–pathological variables including age, TNM stage, and performance status did not differ significantly between patients with high and low expression of ERCC1 (Table 1). The high ERCC1 expression group included more men (*P*=0.022) and more smokers (*P*=0.013, Table 1). Squamous cell carcinoma of the head and neck of the hypopharynx showed higher expression of ERCC1.

### Relationship between treatment response and ERCC1 expression

The overall response rate after CCRT for all patients was 89% (40 out of 45, 27 complete responses and 13 partial responses; 3 with stable disease and 2 with progressive disease). Patients with low expression of ERCC1 achieved a higher complete response (10 out of 12, 83%) compared with 52% (17 out of 33) of patients with high expression of ERCC1, although this was not significant (*P*=0.086, Table 2).

### Relationship between survival and ERCC1 expression

**Figure 2:**
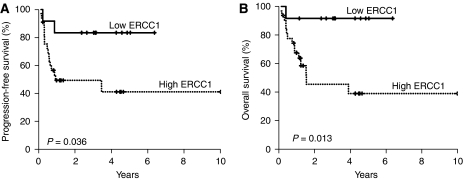
Kaplan–Meier estimates of the probability of survival. (**A**) PFS according to ERCC1 expression. (**B**) OS according to ERCC1 expression.

The median follow-up was 53.6 months (95% CI, 34.5–72.7 months). The overall 3-year PFS rate was 58.7% (95% CI, 44.0–73.4%) and the 3-year OS rate was 61.3% (95% CI, 45.4–77.2%). The 3-year PFS for patients with low expression of ERCC1 was 83.3% (95% CI, 62.1–100.0%) compared with 49.4% (95% CI, 31.8–67.0%) for patients with high expression of ERCC1 (*P*=0.036, Figure 2A). The 3-year OS rate was significantly longer in patients with low expression of ERCC1 (91.7; 95% CI, 76.0–100.0%) than in those with high expression of ERCC1 (45.5; 95% CI, 23.9–67.1%) (*P*=0.013, Figure 2B). The univariate analysis revealed that tumour stage, tumour location, and ERCC1 expression were important factors affecting the prolongation of both PFS and OS (Table 3). The multivariate analysis also showed that low expression of ERCC1 (HR 0.120; 95% CI, 0.016–0.934%) (*P*=0.043) together with tumours other than oral cavity primary tumours (HR 0.168; 95% CI, 0.040–0.707) (*P*=0.015), and stage III tumours (HR 0.081; 95% CI, 0.009–0.716) (*P*=0.024) were independent predictors of the prolongation of OS (Table 4).

## Discussion

We found a high expression of ERCC1 in 73% of SCCHN tumours. The median percentage of ERCC1-stained nuclei was 92%, and the median *H* score was 2.0, values that are consistent with a previous report (Handra-Luca *et al*, 2007). In contrast, in a study of non-small-cell lung cancer (NSCLC), the median *H* score for ERCC1 expression was 1.0 and 30–40% of tumours expressed ERCC1 (Olaussen *et al*, 2006), suggesting that the proportion and pattern of ERCC1 expression varies according to the tumour type. It is of interest that in our study, specimens from men and smokers, and the presence of SCCHN of the hypopharynx showed high expression of ERCC1. These findings await confirmation by prospective studies with large numbers of patients.

Patients with low expression of ERCC1 achieved a higher rate of complete response to CCRT than did those with high expression of ERCC1, although the overall response did not differ significantly. It is noteworthy that ERCC1 expression was associated with a significantly longer PFS and OS. The 3-year PFS for patients with low expression of ERCC1 was 83.3% compared with 49.4% for patients with high expression of ERCC1 (*P*=0.036). The 3-year OS rate was also significantly longer in patients with low expression of ERCC1 than in patients with high expression of ERCC1 (91.7 *vs* 45.5%, *P*=0.013). Multivariate analysis revealed that low expression of ERCC1 was an independent factor associated with a lower risk of cancer death (HR 0.12, *P*=0.043). This result is also consistent with a previous report of an increase in tumour response and prolongation of OS in patients treated by cisplatin-based induction chemotherapy for locally advanced SCCHN (Handra-Luca *et al*, 2007). Although the association between ERCC1 expression and clinical outcomes in patients with SCCHN treated with radiotherapy has not been established, in NSCLC, the level of induced DNA adducts in buccal cells is strongly associated with outcome after definitive concomitant low-dose cisplatin and radiotherapy for stage IIIA/B NSCLC (van de Vaart *et al*, 2000). A relationship between the expression of ERCC1 and tumour response or survival was also reported in oesophageal cancer patients treated with chemoradiotherapy (Warnecke-Eberz *et al*, 2004; Joshi *et al*, 2005). In this context, our findings provide additive evidence that the pretreatment level of ERCC1 in tumour cells is negatively related to the treatment outcome of platinum compounds. The predictive role of pretreatment ERCC1 expression level might be connected with the capacity for DNA damage repair, that is, tumour cells with a more efficient DNA repair capacity can be resistant to cisplatin-based chemotherapy or radiotherapy. Further mechanistic study is needed to confirm this concept.

Quantitative real-time reverse transcription–PCR is commonly used to detect ERCC1 expression (Dabholkar *et al*, 1994; Metzger *et al*, 1998; Shirota *et al*, 2001; Lord *et al*, 2002). Although this method is very sensitive and semi-quantitative, it requires fresh tumour samples. A study to determine whether DNA polymorphism of ERCC1 has predictive value in head and neck cancer patients showed that polymorphic variation in DNA repair genes (XPD and XRCC1, not ERCC1) is a powerful prognostic factor for the response to cisplatin in SCCHN patients (Quintela-Fandino *et al*, 2006). However, this polymorphism is associated mainly with lower rates of translation of the ERCC1 gene, which results in low levels of the protein in nucleus. Immunohistochemistry is a clinically feasible method that can be applied in almost every pathology laboratory despite several limitations, including the use of different antibodies, inter- or intraobserver variation, and the variable cutoff value for ERCC1 positivity (Olaussen *et al*, 2006).

Our study has several limitations. First, the study analysed only 45 out of 60 patients with available tissues, and the analysis was based on a retrospective analysis over a long time. During the study period, advances in radiotherapy techniques, chemotherapeutic agents, and supportive care may have affected the tumour response or survival. Nevertheless, we found no significant difference in survival according to the various chemotherapeutic agents. Second, the primary tumour site was also heterogeneous, and the prognosis of SCCHN is dependent on the primary tumour site: oral cavity cancer has a worst prognosis and laryngeal cancer a good prognosis. We also found that patients with oral cavity cancer had short PFS and OS, each of which represented an independent prognostic factor. Regardless, a high expression of ERCC1 was strongly correlated with poor survival regardless of tumour location. Because our study comprised only a small number of patients for each tumour location, caution should be used when drawing conclusions from our data, which need to be validated prospectively with more homogeneous and a large number of patients.

In conclusion, this study suggests that ERCC1 expression levels negatively contribute to the clinical outcomes including PFS and OS in patients treated with cisplatin-based CCRT for locally advanced SCCHN. This suggests that ERCC1 expression might be a useful predictive marker.

## Figures and Tables

**Table 1:** Characteristics of patients

	**All patients (*N*=45)**	**Low ERCC1 (*N*=12)**	**High ERCC1 (*N*=33)**	
**Characteristics**	**Number (%)**			***P-*value**
*Age (years)*				0.283
⩽60	30 (67)	10 (83)	20 (61)	
>60	15 (33)	2 (17)	13 (39)	
				
*Sex*				0.022
Male	37 (82)	7 (58)	30 (91)	
Female	8 (18)	5 (42)	3 (9)	
				
*T stage*				0.699
T1–T2	11 (24)	2 (17)	9 (27)	
T3–T4	34 (76)	10 (83)	24 (73)	
				
*N stage*				0.743
N0–N1	17 (38)	5 (42)	12 (36)	
N2–N3	28 (62)	7 (58)	21 (64)	
				
*Stage*				1.000
III	8 (18)	2 (17)	6 (18)	
IVA	30 (67)	8 (66)	22 (67)	
IVB	7 (15)	2 (17)	5 (15)	
				
*Location*				0.044
Paranasal sinus	5 (11)	3 (25)	2 (6)	
Oral cavity	5 (11)	1 (8)	4 (12)	
Oropharynx	15 (33)	5 (42)	10 (30)	
Hypopharynx	12 (27)	0 (0)	12 (36)	
Larynx	8 (18)	3 (25)	5 (15)	
				
*Performance status*				0.705
0	12 (27)	4 (33)	8 (24)	
1	33 (73)	8 (67)	25 (76)	
				
*Smoking*				0.013
Yes	35 (78)	6 (50)	29 (88)	
No	10 (22)	6 (50)	4 (12)	
				
*Alcohol*				0.283
Yes	30 (67)	6 (50)	24 (73)	
No	15 (33)	6 (50)	9 (27)	

ERCC1=excision repair cross-complementation group 1.

**Table 2:** Expression of ERCC1 and response to treatment

	**Low ERCC1 patients (*N*=12)**	**High ERCC1 patients (*N*=33)**	
	**Number (%)**		***P*-value**
Response			0.010
Complete response	10 (83)	17 (52)	
Partial response	0 (0)	13 (39)	
Stable disease	2 (17)	1 (3)	
Progression disease	0 (0)	2 (6)	

ERCC1=excision repair cross-complementation group 1.

**Table 3:** Univariate analyses of prognostic factors for survival

	**3-year PFS**		**3-year OS**	
	***N*=45**		***N*=44**	
	**Percent (95% CI)**	***P*-value**	**Percent (95% CI)**	***P*-value**
*Sex*		0.580		0.336
Male	58.2 (41.9–74.5)		58.9 (40.9–76.9)	
Female	62.5 (29.0–96.0)		72.9 (40.6–100.0)	
				
*Age (years)*		0.572		0.687
⩽60	55.9 (37.9–73.9)		60.3 (41.9–78.7)	
>60	65.0 (40.1–89.9)		59.6 (22.4–96.8)	
				
*Smoking*		0.702		0.481
Yes	58.7 (42.0–75.4)		60.2 (42.0–78.4)	
No	60.0 (29.6–90.4)		66.7 (35.1–98.3)	
				
*Stage*		0.002		0.003
III	75.0 (45.0–100.0)		83.3 (53.5–100.0)	
IVA	68.9 (51.8–86.0)		70.7 (53.5–87.9)	
IVB	0		0	
				
*Tumour location*		<0.001		<0.001
Oral cavity	0		0	
Others	66.2 (51.1–81.3)		70.7 (54.8–86.6)	
				
*Performance status*		0.491		0.986
0	50.0 (21.8–78.2)		66.7 (40.0–93.4)	
1	62.0 (44.9–79.1)		60.3 (41.5–79.1)	
				
*ERCC1 expression*		0.036		0.013
High	49.4 (31.8–67.0)		45.5 (23.9–67.1)	
Low	83.3 (62.1–100.0)		91.7 (76.0–100.0)	
				
*Chemotherapy regimen*		0.245		0.151
Cisplatin	46.0 (19.9–72.1)		49.7 (22.8–76.6)	
Fluorouracil+cisplatin	50.0 (10.0–90.0)		50.0 (10.0–90.0)	
Taxane+cisplatin	68.2 (48.8–87.6)		73.9 (53.5–94.3)	
				
*Completion of chemotherapy*		0.192		0.078
Yes	61.6 (45.7–77.5)		65.6 (48.5–82.7)	
No	42.9 (6.2–79.6)		42.9 (6.2–79.6)	
				
*Radiation dose (Gy)*		0.296		0.381
⩽66.6	48.5 (27.9–69.1)		55.3 (34.3–76.3)	
>66.6	70.0 (50.0–90.0)		67.4 (42.3–92.5)	

ERCC1=excision repair cross-complementation group 1; OS=overall survival; PFS=progression-free survival.

**Table 4:** Hazard ratios for progression-free survival and overall survival

	**PFS**		**OS**	
	**HR (95% CI)**	***P*-value**	**HR (95% CI)**	***P*-value**
Location (others *vs* oral cavity)	0.155 (0.051–0.470)	0.001	0.168 (0.040–0.707)	0.015
Stage	NE	0.112		0.076
III *vs* IVB			0.081 (0.009–0.716)	0.024
IVA *vs* IVB			0.575 (0.164–2.010)	0.386
ERCC1 expression (low *vs* high)	NE	0.077	0.120 (0.016–0.934)	0.043
Completion of chemotherapy	NE	0.154	NE	0.348

ERCC1=excision repair cross-complementation group 1; NE=not in the equation; OS=overall survival; PFS=progression-free survival.
